# Cochleo-Vestibular Disorders in Herpes Zoster Oticus: A Literature Review and a Case of Bilateral Vestibular Hypofunction in Unilateral HZO

**DOI:** 10.3390/jcm12196206

**Published:** 2023-09-26

**Authors:** Roberto Teggi, Anna Del Poggio, Iacopo Cangiano, Alessandro Nobile, Omar Gatti, Mario Bussi

**Affiliations:** 1 Otolaryngology Department, San Raffaele Scientific Institute, 20132 Milan, Italy; cangiano.iacopo@hsr.it (I.C.); nobile.alessandro@hsr.it (A.N.); gatti.omar@hsr.it (O.G.); bussi.mario@hsr.it (M.B.); 2 Department of Neuroradiology and CERMAC, San Raffaele Hospital, 20132 Milan, Italy; delpoggio.anna@hsr.it

**Keywords:** herpes zoster oticus, Ramsay Hunt syndrome, facial palsy, vestibular disorders, hearing loss, bilateral cochleo-vestibular damage, magnetic resonance imaging

## Abstract

The varicella-zoster virus (VZV), a member of the Herpesviridae family, causes both the initial varicella infection and subsequent zoster episodes. Disorders of the eighth cranial nerve are common in people with herpes zoster oticus (HZO). We performed a review of the literature on different databases including PubMed and SCOPUS, focusing on cochlear and vestibular symptoms; 38 studies were considered in our review. A high percentage of cases of HZO provokes cochlear and vestibular symptoms, hearing loss and vertigo, whose onset is normally preceded by vesicles on the external ear. It is still under debate if the sites of damage are the inferior/superior vestibular nerves and cochlear nerves or a direct localization of the infection in the inner ear. The involvement of other contiguous cranial nerves has also been reported in a few cases. We report the case of a patient with single-side HZO presenting clinical manifestations of cochleo-vestibular damage without neurological and meningeal signs; after 15 days, the patient developed a new episode of vertigo with clinical findings of acute contralateral vestibular loss. To our knowledge, only three other such cases have been published. An autoimmune etiology may be considered to explain these findings.

## 1. Introduction

Varicella-zoster virus (VZV), a member of the Herpesviridae family, causes both the initial varicella infection and subsequent zoster episodes. Herpes zoster oticus (HZO) is characterized by otalgia—vesicular eruptions of the auricle or external auditory canal—and oral mucosa and the entire pharyngeal column may sometimes be involved [1]. Ramsay Hunt syndrome (RHS) can be diagnosed when ipsilateral facial nerve palsy coexists [2,3]. Disorders of the eighth cranial nerve are common in people with HZO, and cochlea-vestibular symptoms such as tinnitus, hearing loss, and vertigo are reported in a significant portion of patients [4]. In addition, paralysis of other cranial nerves has been reported in subjects with RHS [5,6]. The purpose of this work was to review the current literature on cochleo-vestibular damage in HZO, with particular focus on vestibular disorders; moreover, we present a case of unilateral HZO with evolution in a few days toward bilateral cochlea-vestibular involvement.

## 2. Materials and Methods

We performed a review of the literature on different databases including PubMed and SCOPUS according to the “Preferred Reporting Items for Systematic Reviews and Meta-analyses” (PRISMA) guidelines.

**Figure 1:**
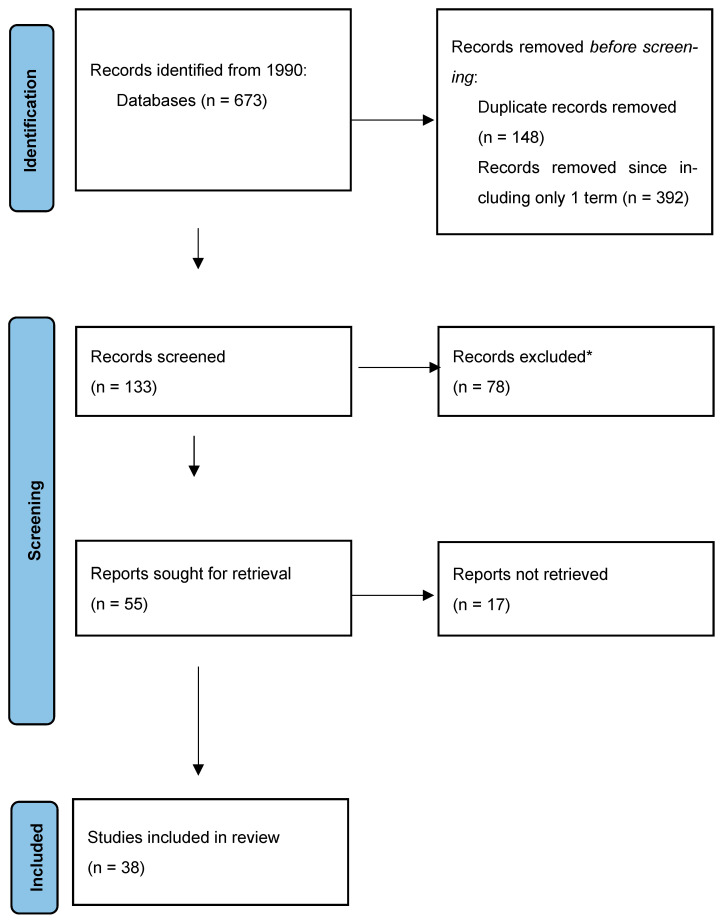
Search strategy flowchart. * Publications citing HZO but not focused strictly on the argument and not presenting new data were excluded.

We used the following search terms: “Herpes Zoster Oticus”, “Herpes Zoster Oticus and Audiovestibular Disorders”, and “Ramsay Hunt syndrome and audiovestibular disorders”. A total of 673 original articles were found. Only papers with both keywords Dizziness\Vestibular Impairment\Balance Disorders on one side AND Elderly on the other were included (see Figure 1).

## 3. Results

After initial clinical manifestations in the auricle, vertigo and hearing loss are commonly reported in patients with HZO [7]. It is commonly accepted that reactivation of VZV in the ganglion of the seventh cranial nerve may cause RHS; the typical symptoms of the eighth nerve, namely hearing loss and vertigo, may be due to spreading via vestibulo-facial or vestibulo-cochlear anastomosis. This hypothesis is supported by the identification of VZV-DNA in both the geniculate ganglion and the vestibular and cochlear nerves [8,9,10]. Since, in some cases, an involvement of the cochlea and vestibule has been found, transmission of the virus to the inner ear has been postulated from the dehiscent facial canal through the oval or round window [11].

Different authors have reported the involvement of the cochlear and retrocochlear systems in a range between 8% and 85%, and hearing loss is frequently more severe at high frequencies and in subjects reporting vertigo [12,13,14,15]. When hearing loss and vertigo are present, facial palsy is almost always associated [1]. The wide range reported may be justified by the different inclusion criteria for RHS and the means of investigation. Kaberos et al. [16] reported hearing loss in 12 of 15 patients. They also investigated acoustic brainstem responses and transiently evoked otoacoustic emissions; when also considering these tests, only one patient showed no cochlear/retrocochlear damage. In particular, 8 patients presented signs of retrocochlear involvement; 3 of these showed a pure retrocochlear pattern, while 3 presented a pure cochlear pattern. In the same publication, the authors reported that 11 of 15 patients had vestibular symptoms [16]. Similarly, by performing extensive cochlear tests, Wayman et al. found that 6 of 7 patients had audiological findings that supported a cochlear pattern [14].

Vestibular disorders in RHS have been reported more frequently, in 50–80% of cases [13,17]. The different means of investigation (i.e., only caloric tests or a larger number of exams) may justify this wide range. Huang et al. reported that in a sample of 20 subjects, only 40% showed abnormal calorics, while 65% presented pathological cervical vestibular-evoked myogenic potentials (cVEMPs) [17]. Conversely, the authors found that involvement of the eighth nerve was prognostic for poor recovery of facial palsy [18].

Martin-Sanz et al. reported that, when present, the vestibular damage evaluated using a video head impulse test often presented a lower gain of the vestibulo-oculomotor reflex compared to patients with vestibular neuritis; this finding may support the hypothesis that both disorders may be due to an eighth nerve disorder of viral origin [19]. Similar results have been reported by Kim et al.; they used skull vibration and a hyperventilation test in a sample of subjects with RHS and compared the results with those of patients with vestibular neuritis and sudden sensorineural hearing loss with vertigo. Skull vibration was positive in around 90% of patients in both cohorts, while hyperventilation, suggestive for a nerve lesion, was detected in 90% of patients with RHS and vestibular neuritis but in only 59% of subjects with sudden sensorineural hearing loss with vertigo. It was concluded that the lesioned site may be more likely within the vestibular nerve than in the inner ear [20].

A case has been reported in which vestibular disorders preceded RHS manifestations, since vertigo preceded the appearance of vesicles by 3 days [21].

Interestingly, Saito et al. reported two cases of RHS with canal paresis and hearing loss; one had normal VEMPs, suggesting only involvement of the superior vestibular nerve [22].

Damage to the inner ear does not necessarily affect cochlear and vestibular function with the same frequency. Takahashi et al., in a sample of 19 patients, found that abnormal tests were demonstrated in 79% by calorics, 53% by oVEMPs, and 17% of cVEMPs, while 26% presented refractory hearing loss. They also performed an MRI with images reconstructed perpendicular to the internal auditory canal to identify the superior and inferior vestibular nerves and the cochlear nerve. The signal intensity increase of the four-nerve enhancement was calculated. An overlap between abnormal tests studying the inferior (cVEMPs)/superior vestibular (calorics mainly) and cochlear nerve was found [23]. Similar results were reported by Ozeki et al.; in 10 subjects with RHS, they found abnormal calorics in all, but cVEMPs were absent in 7 patients. In four patients, they also performed VEMPs with galvanic stimulation (which stimulates directly the inferior nerve) in order to establish the site of the lesion; two presented no VEMPs, supporting the hypothesis of a lesion of the inferior vestibular nerve, while two presented normal responses, addressing the diagnosis toward a lesion of the saccule [24].

Almost all studies reported a nystagmus beating toward the unaffected side in patients with RHS related to a vestibular deficit of the pathological side [22,25]; when mapping vestibular nerve function, both seemed to be equally affected [26].

Lee et al. studied the presence of nystagmus in RHS with and without dizziness; in 96% of patients reporting vestibular symptoms, they found a spontaneous nystagmus, while in 67% of patients without dizziness, nystagmus was of weaker intensity [27]. The presence of vertigo and nystagmus does not seem to be related to viral load, since no significant difference in maximum viral copy number between patients with and without cochlea-vestibular symptoms was found in the saliva of patients with RHS [28]. Finally, Kim et al. reported a change in bipositional nystagmus direction (both geotropic and apogeotropic) in 7 of 28 patients; according to the authors, this finding may be, at least in part, explained by the alteration in specific gravity of the lateral semicircular canal cupula or endolymph due to inflammation in the inner ear membrane [29]. Results of cochlear and vestibular tests in patients with RHS are reported in Figure 1.

While a lesion of the 7th–8th cranial nerves is commonly found, different cases have been published reporting involvement of the 9th–12th cranial nerves in RHS; it has been hypothesized that the mechanisms involved in the pathogenesis of cranial polyneuropathy may include the direct perineural and trans-axonal spread of viral inflammation between contiguous cranial nerves and hematogenous dissemination between nerves with shared blood supply [6,15,30]. 

As previously reported, studies trying to assess the site of lesions leading to vertigo and hearing loss are often controversial, and the question of whether the inferior or superior vestibular nerves are involved more frequently or if the inner ear is also involved is still under debate. Interesting results may be drawn from recent studies through gadolinium-enhanced magnetic resonance imaging (MRI). Berrettini et al. [31] in four cases found a good correlation between clinical examination and 7th–8th nerve enhancement; this underlines that the cochleo-vestibular damage observed may be due to the nerve rather than to a direct localization in the inner ear. Overlapping results have been reported by Iwasaki et al. [32]; they tried to selectively identify vestibulocochlear nerves in the internal auditory canal using a gadolinium-enhanced 1.5 Tesla MRI in a sample of 14 subjects and correlated the findings with calorics and audiometric exams. All patients except one with canal paresis exhibited enhancement of the superior vestibular nerve, thus demonstrating a good association between clinical and imaging data. On the other hand, 7 of 10 patients with hearing loss showed no cochlear nerve enhancement; these patients showed faster and complete recovery from hearing loss. The authors concluded that only refractory hearing loss might be associated with enhancement of the nerve [31].

Different results have been reported by Lee et al. who studied inner ear structures in 18 patients with a 4 h post-contrast 3D-fluid-attenuated inversion recovery sequence (3D-FLAIR) 3-Tesla MRI and correlated the results with clinical data. In these patients, 13 showed high signal intensity of the inner ear endorgan on the lesion side compared to the unaffected side. Twelve patients showed abnormal calorics, nine of whom showed high signal intensity of LSCC in post-contrast 3D-FLAIR, while six subjects had pathological results of the video head impulse test. Of the 12 patients with hearing loss, 9 showed high signal intensity of the cochlea in post-contrast 3D-FLAIR [32]. 

As a final consideration, both vestibular nerves and the inner ear may be the sites of infectious localization and viral infection may spread to other contiguous cranial nerves. In the Table 1 results of the most important published studies have been summarized.

Below, we present the case of a patient with a single-side HZO with clinical manifestations of bilateral cochlea-vestibular damage; to our knowledge, only three other such cases have been published.

### Case Report

**Figure 2:**
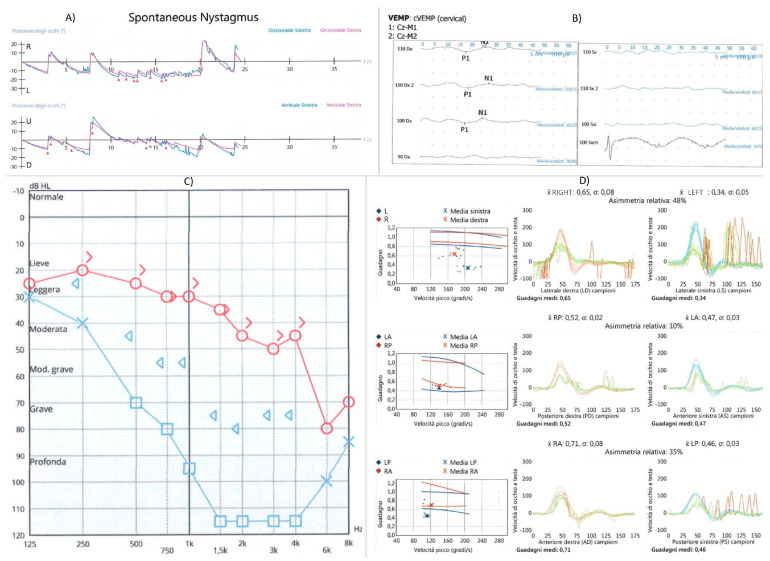
Spontaneous nystagmus (**A**), cervical VEMPs (**B**), audiometric exam (**C**), and video head impulse test (**D**) at first evaluation showing left cochleo-vestibular damage.

A 66-year-old woman was admitted to our hospital with gradually increasing pain in the left ear and sore throat in the last 5 days. She had no vertigo, nausea, facial palsy, or hearing loss. Upon ear, nose, and throat examination, multiple vesicles and scabs were visible on the left auricle and external ear canal; in oro-laryngoscopy of the anterior and posterior left tonsil column, the presence of multiple coalescent erosive lesions was visible extending to the entire pharyngeal column and epiglottis up to the ipsilateral pyriform sinus. A CT scan performed at admission showed no lesions in the brain or ear. Blood tests showed a significant increase in inflammatory markers and viral DNA research for VZV was positive (2,500,000 copies/mL). Three days after hospitalization, the patient began to feel nauseous and reported hearing loss and vertigo; she had a negative brain MRI without contrast that was performed to rule out an acute ischemic event or meningitis. Vestibular evaluation showed a spontaneous nystagmus beating toward the right side that was partially inhibited by fixation and increased while lying on the left side. A video head impulse (Otometrics) showed a reduced left VOR gain for all the three canals, and c-VEMPs were absent. The audiometric test showed severe hearing loss on the left side, mainly at high frequencies, and mild sensorineural hearing loss on the right side. Results of the audiometric exam and video head impulse are summarized in Figure 2. 

**Figure 3:**
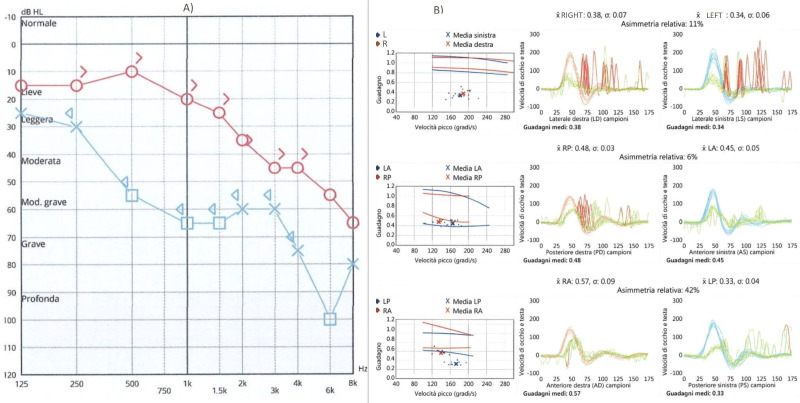
Results of audiometric exam (**A**) and video head impulse test (**B**) at the onset of contralateral vestibular damage. Right side in red, left side in blue. Audiometric exam did not show changes, while bilateral vestibular damage was found.

The patient was treated with steroids and acyclovir and was released from the hospital after 12 days with clinical and laboratory improvement. Ten days after discharge, the patient presented to the Emergency Room for difficulty walking, ataxia, and severe dizziness. She had a left beating nystagmus and a video head impulse test showed a reduced gain of the vestibulo-oculomotor reflex bilaterally and symmetrically; all six semicircular canals were involved. Neurological evaluation was negative; she did not present meningeal signs. The patient was hospitalized for therapy and further exams. Results of the video head impulse and audiometric exam are shown in Figure 3.

**Figure 4:**
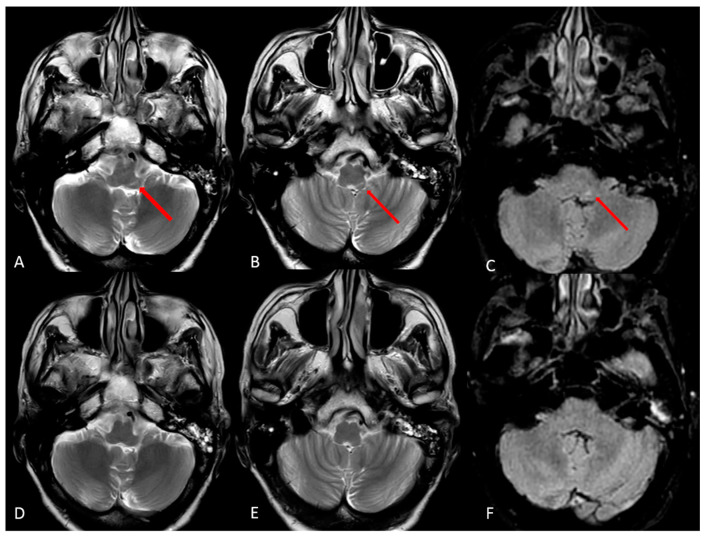
(**A**–**C**) MRI scan with contrast of the patient after the second hospitalization; (**D**–**F**) MRI scan without contrast performed during the first hospitalization. (**A**,**B**,**D**,**F**) T2-weighted images; (**C**,**F**) FLAIR images. (**A**–**C**) Hyperintense lesion (red arrows) in the posterior lower pons on the left side extended to the medulla, in the presumed side of cochlear and vestibular nuclei.

**Figure 5:**
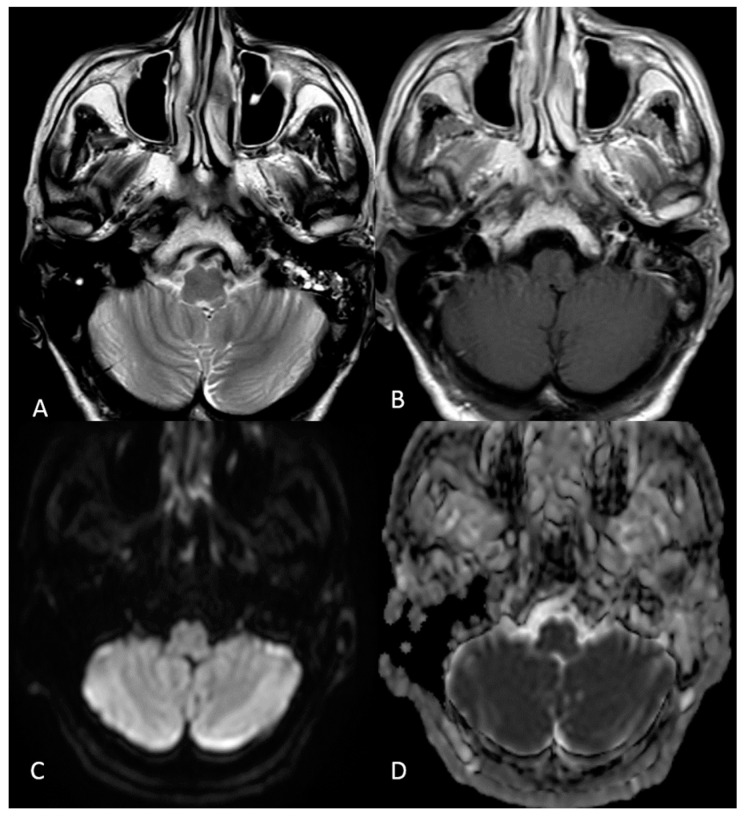
(**A**) T2-weighted image; (**B**) T1-weighted image post-contrast administration; (**C**,**D**) diffusion-weighted imaging b-1000 and apparent coefficient diffusion map, respectively. The lesion lacked contrast enhancement (**B**) and there was no diffusion restriction of water within the alteration (**C**,**D**).

A contrast MRI was performed at 19 days after the previous one. On imaging, there was the presence of a linear signal alteration on the left side of the posterior lower pons extended to the medulla in the presented site of cochlear and vestibular nuclei. The lesion did not show enhancement or diffusion restriction and lacked any activity features (Figure 4 and Figure 5). It was not typical of a recent ischemic lesion, because of the lack of vascular territories affected and no restricted diffusion within the alteration. Regarding medical history, a possible post-infectious significance without signs of disease activity was presumed.

Seven days after the second hospitalization, the clinical conditions improved with no spontaneous nystagmus; the patient was released, although they still had ataxic march. 

In all video head impulse tests, OVERT corrective saccades were recorded.

Two months later, the video head impulse showed signs of partial recovery of both vestibulo-oculomotor reflexes, although worse vestibular function was recorded on the right side. Moreover, almost complete recovery of hearing loss on the left was demonstrated. The audiometric exam was similar to a previous test performed elsewhere demonstrating presbycusis.

**Figure 6:**
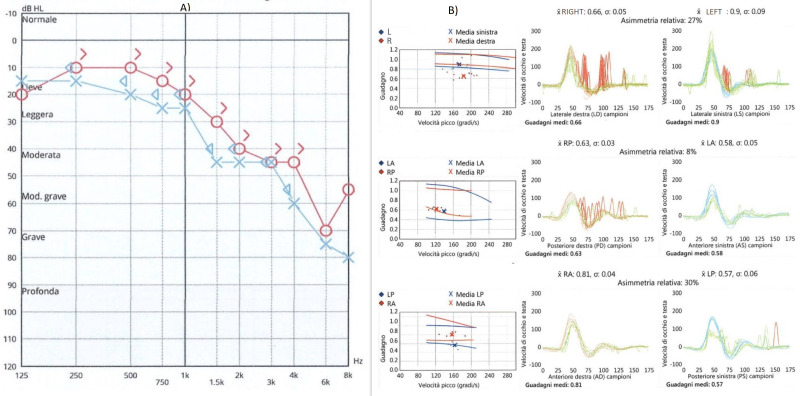
Audiometric exam (**A**) and video head impulse test (**B**) performed at 2 months after discharge.

Results of the vestibular tests and audiometric exam are shown in Figure 6.

## 4. Discussion

As previously stated, both inferior/superior vestibular and cochlear nerves may be damaged by HZO and viral infection may spread to other contiguous cranial nerves. It is still under debate if cochleo-vestibular damage may be more often due to inferior/superior nerve or inner ear localization of the virus; both mechanisms probably play a role. For this purpose, new clinical tests and, above all, MRI demonstrating enhancement may address this aspect in the future.

Among the pathophysiological mechanisms provoking cranial polyneuropathy, the direct perineural and trans-axonal spread of viral inflammation between contiguous cranial nerves and the hematogenous dissemination between nerves with a shared blood supply have been proposed.

The case we present is characterized by HZO on one side; the patient developed a contralateral vestibular deficit after 10 days without signs of HZO; steroids and antiviral therapy were effective in both vertigo attacks. To our knowledge, only three other such cases have been published [33,34,35]. A hematogenous dissemination seems unlikely. In the present case, since a contiguous infection seems improbable, the most convincing in our opinion is that proposed by Schulz et al. [34] that the contralateral loss of vestibular function could result from an autoimmune mechanism. 

Some clinical features in our opinion underline this possibility, which is supported in the case of Schulz et al. by the presence of autoimmune autoantibodies toward the brain area. The patient had a complete resolution of vesicular eruptions in the left ear and anti-VZV antibodies had dropped to very low levels. The onset of the contralateral vestibular deficit occurred without vesicles on the auricle and no meningeal signs were present. These considerations were supported by imaging, where an alteration with no sign of disease activity was located in the presumed site of the contralateral cochlear and vestibular nuclei. The hematogenous spread of the infection was more unlikely, considering the lack of contrast enhancement of the cisternal component of the cranial nerves involved.

The association of infection with herpes virus family and autoimmune disorders has been proposed for other conditions, including herpes ophthalmicus and Guillan–Barrè syndrome [36,37,38].

An autoimmune mechanism has also been proposed in a previously published report in which the onset of contralateral vestibular deficit occurred 5 months after the HZO [33]. The authors supported their hypothesis by performing an immunofluorescence test with the patient’s serum, which showed the presence of autoantibodies having a cross-reaction toward the cerebellum and brain and weaker toward a rat nerve; no reaction was demonstrated for other rat organs.

The lack of an immunological test may be considered as a potential limitation in our case. Nonetheless, it should be considered that the lack of further information is related to the fact that the case dates to 1994 when additional tests were not available. The timing of the onset of contralateral vestibular deficit in our case was more rapid but compatible with an autoimmune etiology. The video head impulse test gave us the possibility to quantify the vestibular deficits. Moreover, contrast MRI demonstrated post-infectious signs of disease only in the ear affected by the primary infection, but were absent in the contralateral side during the onset of the second vestibular loss, excluding a contralateral localization of the virus.

## Figures and Tables

**Table 1:** Results of cochlear and vestibular tests in patients with RHS; case reports are not included.

	N.	Exams Performed	Main Findings
Wayman, 1990 [14]	7	Audiometry, ABR	6 presented cochlear pattern
Kaberos, 2002 [16]	15	Audiometry, ABR, Otoacoustic emissions	12 presented hearing loss 8 with retrocochlear involvement 3 with pure cochlear hearing loss
Huang, 2015 [17]	20	Caloric tests, c VEMPs	8 abnormal calorics 13 pathological VEMPs
Kim, 2015 [20]	17	Vestibular tests	Positive skull vibration in 94% Hyperventilation-positive in 59%
Saito, 2003 [22]	2 with canal paresis	Caloric tests, c VEMPs	1 with pathological VEMPs
Takahashi, 2021 [23]	19	Caloric tests, o and c VEMPs	79% pathological caloric tests 53% pathological o-VEMPs 17% pathological c-VEMPs
Ozeki, 2006 [24]	10	Caloric tests, c VEMPs	10 abnormal caloric tests 7 pathological VEMPs
Lee, 2021 [27]	36; 27 with and 9 without dizziness	Vestibular tests	67% of subjects without dizziness presented spontaneous nystagmus
Kim, 2018 [29]	28	Vestibular tests	7 of them presented a bipositional direction changing nystagmus

## Data Availability

Ethical review and approval were waived for this study since it reports a single case; the patients performed only exams for the clinical condition and no extra exams were performed for research.
